# The association of alcohol use and positive and negative urgency to same day objective binge eating in emerging adults

**DOI:** 10.3389/fpsyg.2023.1152691

**Published:** 2023-08-23

**Authors:** Sarah Fischer, Joseph Wonderlich, Leo A. Miller, Lauren Breithaupt, Rachel Frietchen, Li Cao, Jillian D. Nelson, Alyssa Izquierdo

**Affiliations:** ^1^Department of Psychology, George Mason University, Fairfax, VA, United States; ^2^Eating Disorders and Weight Management Center, Sanford Health, Fargo, ND, United States; ^3^Department of Psychiatry, Massachusetts General Hospital and Harvard Medical School, Boston, MA, United States; ^4^Butler Hospital, Brown University, Providence, RI, United States

**Keywords:** positive urgency, binge eating, alcohol, emerging adults, EMA

## Abstract

**Introduction:**

Objective binge eating and problematic alcohol use often co-occur and are common behaviors in emerging adults. Both behaviors are thought to be driven by affect regulation processes. Objective binge eating often occurs in the context of increasing or acute negative affect, and often occurs in solitude. Alcohol use in emerging adults can also be associated with negative affect regulation. However, in contrast to objective binge eating, a large body of research indicates that there are positively valenced pathways to alcohol use in this age group. Emerging adults often drink socially, to enhance enjoyment, and in the context of positive mood. We propose that one pathway to objective binge eating in this developmental period is through alcohol use itself, such that emerging adults who consume alcohol and who are more likely to act impulsively in the context of positive emotion (i.e., have high levels of positive urgency) may be more likely to binge eat following drinking.

**Methods:**

We collected data using ecological momentary assessment in 106 undergraduates on positive and negative affect, motives for drinking and eating, and alcohol use and objective binge eating, in addition to baseline questionnaires of impulsivity.

**Results:**

There were no significant changes in affect prior to drinking in this sample. Alcohol use at one time point significantly increased odds of objective binge eating at a later time point in the same day. Individual differences in positive urgency, the tendency to act rashly while experiencing positive affect, were also associated with increased odds of objective binge eating that occurred after alcohol use. Individual differences in negative urgency, the tendency to act rashly after experiencing negative affect, did not have a main effect on objective binge episodes, but did interact with alcohol use to increase the odds of objective binge eating following drinking. The vast majority of drinking episodes prior to objective binge eating were social drinking episodes, and participants most commonly endorsed "to have fun" as a reason for drinking.

**Discussion:**

Results suggest that alcohol consumption may increase risk for objective binge eating in emerging adults.

## Introduction

Eating disorders (EDs) and alcohol use disorder (AUD) frequently co-occur. AUD is a condition in which individuals experience symptoms related to loss of control over alcohol use and associated negative consequences, and may be mild, moderate, or severe (American Psychiatric Association, 2022). Objective binge eating, defined as eating an objectively large amount of food with associated loss of control, may be present in individuals within multiple different ED diagnoses [anorexia nervosa (AN), bulimia nervosa (BN), binge eating disorder (BED), or otherwise specified feeding and eating disorder (OSFED)] (American Psychiatric Association, 2022).

Problematic alcohol use often co-occurs with binge eating symptoms (e.g., Mason et al., 2021) in both clinical samples and emerging adult samples. For example, rates of lifetime AUDs in individuals with BED are significantly higher than in individuals without BED (Bogusz et al., 2021). And, rates of EDs are higher in women with AUD or nicotine dependence than women without these disorders (Munn-Chernoff et al., 2020). Similarly, objective binge eating and other disordered eating symptoms commonly co-occur with problematic alcohol consumption (such as binge drinking) in emerging adults and undergraduate students (Nelson et al., 2009; Trojanowski et al., 2019), and both behaviors may onset prior to the development of a full diagnosis of an AUD or ED.

Emerging adulthood, the period of time between the end of compulsory schooling and adult commitments (Arnett, 2000, 2007), is a high risk period of time for the development and maintenance of these behavior patterns, as well as other risky behaviors (Gates et al., 2016; Goldschmidt et al., 2016; Schwartz and Petrova, 2019). Recent studies indicate a prevalence rate of 7.6% for objective binge eating in first year college students, and a rate of 49.3% past month alcohol use in young adults age 18–22 (Serra et al., 2020; National Survey on Drug Use Health, 2021). Individuals in this developmental stage often experience instability, identity self-exploration, self-focus, and transitions between romantic partners, career choices, and other life choices (Arnett, 2007). During this time of less structure and more transition, there are fewer constraints on impulse control. Thus, maladaptive behaviors, such as objective binge eating, may become habitual over this developmental period (e.g., Goldschmidt et al., 2016). As such, emerging adulthood is an important developmental period to study mechanisms through which comorbidity may develop.

### Affect, alcohol use, and binge eating

Several pathways have been proposed to explain the link between objective binge eating and alcohol use (Ferriter and Ray, 2011; Escrivá-Martínez et al., 2020). One commonly studied hypothesis is that binge eating and alcohol use may both function to regulate acute negative affect (Baker et al., 2004; Pearson et al., 2015). For example, individuals who both binge eat and binge drink endorse negative affect regulation motives for these concurrent behaviors (Trojanowski et al., 2019). The affect regulation model of binge eating posits that food serves as a concrete stimulus to distract attention away from aversive self-criticism or negative affect. Binge eating is maintained via negative reinforcement, as it quickly reduces acute negative emotion. In support of this hypothesis, EMA studies have documented that negative affect increases prior to objective binge eating and decreases after objective binge eating (e.g., Smyth et al., 2007; Berg et al., 2013; Fischer et al., 2018). Similar models have been described in the alcohol use field. The negative reinforcement model of drinking posits that individuals drink after perceiving cues for the onset of negative emotion (Baker et al., 2004). This pattern is negatively reinforced and individuals then start to drink following the experience of negative affect because they have developed expectancies that alcohol will alleviate distress (Baker et al., 2004). Studies utilizing EMA to examine temporal patterns of emotion and alcohol use to test this hypothesis have mixed results, with some studies documenting that negative affect precedes drinking while others do not (Simons et al., 2005).

Although both objective binge eating and drinking tend to onset in adolescence or early adulthood and have similar pathways, there are important ways in which they differ (Birch et al., 2007). Objective binge eating characteristic of EDs often occurs in isolation and in states of negative emotion or stress (e.g., Birch et al., 2007; Smyth et al., 2007). However, many emerging adults tend to drink in order to enhance positive mood states, when socializing, or when in a positive mood (Creswell, 2021). Solitary drinking, in contrast, is often associated with negative affect (Creswell, 2021). Two ecological momentary assessment (EMA) studies have found that daily experience of both negative and positive affect are predictive of alcohol consumption (Simons et al., 2005, 2010). Thus, there are positive and negatively valenced pathways to drinking for a large proportion of emerging adults who engage in this behavior (Creswell, 2021). In contrast, there is a dearth of data on positive emotional experiences or enhancement motives on objective binge eating. Some experimental studies suggest that individuals snack more when experiencing positive mood or consume more calories in a positive mood compared to a neutral mood (Bongers et al., 2013; Evers et al., 2013). However, the limited data on the relationship between positive affect and objective binge eating suggests that positive affect decreases prior to binge eating (Smyth et al., 2007) and that increases in positive affect are prospectively associated with decreases in binge eating (De Young et al., 2014).

### Urgency, alcohol use, and binge eating

Urgency, the tendency to act rashly while experiencing acute affect, is associated with both drinking and objective binge eating (Cyders and Smith, 2008; Berg et al., 2015; Smith and Cyders, 2016). This personality trait is a facet of the broader construct of impulsivity (Whiteside and Lynam, 2001). Whiteside and Lynam (2001) identified four traits related to impulse control described in multiple structural trait models of personality: (lack of) planning, (lack of) persistence, sensation seeking, and urgency. Urgency has two components. One is the tendency to act impulsively while experiencing acute negative affect [negative urgency (NU)], and the other is the tendency to act impulsively while experiencing acute positive affect [positive urgency (PU)] (Cyders et al., 2007; Cyders and Smith, 2008). High levels of negative urgency may increase the likelihood that individuals will down regulate negative emotions with food and/or alcohol (Fischer et al., 2012; Pearson et al., 2015), while high levels of positive urgency are associated with increased alcohol use and other reward seeking behavior (Smith and Cyders, 2016). When individuals act more impulsively while experiencing mood states, they may be more likely to choose behaviors with immediately reinforcing properties. In this way, individual differences in this trait may increase vulnerability to the cycle of affect regulation with food and substances.

Several studies support this hypothesis. NU is robustly and positively associated with binge eating (Fischer et al., 2008; Culbert et al., 2015). Additionally, NU is positively associated with both binge eating and symptoms of AUD (Berg et al., 2015). Individual differences in NU may both increase risk for both binge eating and problems with alcohol, and maintain these problems (Fischer et al., 2012; Pearson et al., 2015). For example, individual differences in NU are associated with increases in both objective binge eating and problem drinking across the first year of college (Fischer et al., 2013; Stojek and Fischer, 2013). PU is robustly associated with alcohol use (Coskunpinar et al., 2013). Individual differences in positive urgency are associated with increased quantity of alcohol consumed per drinking occasion, and when in positive emotional states (Cyders et al., 2010; Dinc and Cooper, 2015). EMA data suggests that PU is more strongly related to drinking to enhance positive emotions and prediction of daily alcohol use than other impulsivity variables (Dora et al., 2022). Prospective studies suggest that first-year college students who rank high in PU are more likely to increase the quantity of alcohol they consume (Cyders et al., 2009).

Despite the fact that PU and NU are highly correlated and represent rash action while experiencing emotion, PU has low to null correlations with objective binge eating (e.g., Kenny et al., 2019). However, some studies suggest that personality traits related to impulse control and positive emotion enhancement, such as reward responsiveness and fun-seeking, are associated with binge eating (Schell et al., 2019). Schell and colleagues found that these traits were indirectly associated with binge eating via the expectancies that eating is rewarding and alleviates boredom.

### Drinking episodes: a possible pathway to binge eating

We suggest that one potential pathway to the establishment of objective binge eating in emerging adults is via the tendency to act impulsively under the influence of alcohol. Alcohol use peaks during this developmental period (e.g., Jochman and Fromme, 2010; Gates et al., 2016). The consumption of alcohol itself contributes to impulsive decision making, increased urges to consume more alcohol, and deficits in inhibitory control (Rose and Duka, 2007; Caswell et al., 2013; Bartholow et al., 2018; Baines et al., 2019). Several methods have been used to examine the dysregulation of food intake associated with alcohol. Meta-analyses, longitudinal studies, and correlational studies indicate that overall caloric consumption and weight gain is higher among people who drink alcohol compared to those who do not (Yeomans, 2010). A variety of studies suggest that individuals who drink do not restrict or cut back caloric consumption after drinking, and thus overall consume more calories in both alcohol and food (Kwok et al., 2019). There are two primary mechanisms through which this may occur, based on experimental studies of the acute effects of alcohol on variables related to food consumption (Rose et al., 2015). First, it is hypothesized that alcohol consumption stimulates the reward system, which increases drive to consume more alcohol or food (Rose et al., 2015). Experimental data to date suggests that this may indeed be the case. Consumption of moderate priming doses of alcohol increases hunger ratings, urges to snack, attentional bias toward food, and overall increase in caloric consumption (Hofman and Friese, 2008; Yeomans, 2010; Rose et al., 2015; Gough et al., 2021). Second, alcohol may impair inhibitory control over eating (Rose et al., 2015). It is difficult to disentangle the impact of increased reward seeking from inhibitory control in the context of studies examining the consumption of food. However, studies of the impact of moderate doses of alcohol on cognitive and motor tasks that assess inhibitory control suggest that these doses increase errors and speed on these tasks (Rose and Duka, 2007). Thus, it is plausible that decreases in inhibitory control may also contribute to overconsumption of food after drinking.

It is important to note that none of these experimental studies assessed objective binge eating or the subjective experience of loss of control. Some EMA studies have assessed how the context of alcohol consumption may impact eating behavior, including objective binge eating. One EMA study of a clinical sample of individuals with BED examined how contextual variables impacted objective binge episodes, including the presence of alcohol (Wilkinson et al., 2022). In this study, for individuals with BED who were also considered heavy drinkers, the presence of alcohol increased the odds of an objective binge episode (Wilkinson et al., 2022). However, this study did not assess alcohol consumption. Another EMA study in a community sample of adults with obesity yielded different findings (Goldschmidt et al., 2014). In that study, whether or not the participant consumed alcohol did not have a significant impact on the likelihood of an objective binge episode (Goldschmidt et al., 2014).

Given that alcohol appears to increase reward seeking and decrease inhibitory control, individual differences in personality variables associated with impulsivity, such as PU and NU, may amplify this effect. Studies utilizing functional magnetic resonance imaging (fMRI) suggest that PU is associated with general response inhibition deficits in individuals with problem drinking (Zandbelt and Vink, 2010; Tervo-Clemmens et al., 2017). To date, neuroimaging and behavioral studies indicate that high levels of trait urgency, positive or negative, are associated with deficits in response inhibition while experiencing emotional arousal (Johnson et al., 2020). Thus, it is possible that high levels of urgency may exacerbate the disinhibiting effects of alcohol consumption on behavior. Specifically, if an individual has reduced ability to inhibit responses because of high levels of urgency, and also consumes alcohol, they may be even less able to inhibit urges to consume food than when not drinking or in comparison to others with low levels of this trait.

### Current study

We propose that one pathway to the development of co-occurring objective binge eating and alcohol use disorder symptoms in the period of emerging adulthood is that the consumption of alcohol itself increases the likelihood of objective binge eating following drinking episodes. We also hypothesize that individual differences in NU and PU are associated with the likelihood of objective binge eating following drinking, and that these individual differences moderate the impact of alcohol use on objective binge eating. We specifically hypothesize that higher levels of both NU and PU will increase the odds of objective binge eating when alcohol use has also occurred. Theoretically, increases in negative affect may be associated with increased likelihood of drinking, and individuals with high NU may be more likely to drink following increases in negative affect. Vulnerability to objective binge eating may increase following this drinking episode, due to both the impact of alcohol itself and the tendency of individuals with high NU to act impulsively to alleviate distress. Alternatively, changes in positive affect may be associated with increased likelihood of drinking, and individuals with high levels of PU may be more likely to drink to amplify or enhance positive emotions following these changes. Vulnerability to objective binge eating may increase following drinking, as described above, due to the disinhibiting impact of alcohol on eating behavior.

In order to test these hypotheses, we conducted a series of analyses using data collected via EMA to reduce retrospective recall bias and examine binge eating, and alcohol use in participants' everyday lives (Smyth and Stone, 2003; Shiffman et al., 2008). We examined the temporal relationship of alcohol use to binge eating using this data.

## Method

### Participants

Participants were 106 individuals (81 women and 25 men) recruited from two universities in the Washington DC area. Individuals were recruited for two different studies, both of which utilized the same EMA protocol and design. Their EMA data was pooled for the purpose of this analysis. Inclusion criteria included access to a smartphone to allow for completion of the phone surveys. As one of the parent studies utilized functional magnetic imaging (fMRI) and the other parent study utilized EEG, exclusion were a psychotic or neurological disorder, history of traumatic brain injury, and left-handedness. On average, participants were 20.75 years old (SD = 3.57; range = 18–43; modal age = 19). Among the participants, 50% (53) identified as White, not Hispanic, 18.87% (20) identified as White, Hispanic, 16.04% (17) as Asian, 6.60% (7) as another ethnicity, 5.66% (6) as Black, Not Hispanic, 2.83% (3) as Black, Hispanic.

### Measures

#### Baseline assessment

##### Positive and negative urgency

PU and NU were assessed with the UPPS-P Impulsive Behavior Scale (UPPS-P; Cyders et al., 2007). The UPPS-P Impulsive Behavior Scale (59 Items) has demonstrated validity and reliability within multiple populations including young adult and undergraduate samples (Cyders and Smith, 2007), clinical samples (Jacob et al., 2010), and adolescent samples (Kim et al., 2022). The PU and NU subscales each consist of 12 items. Individuals rated each item on a 4-point scale ranging from 1 (*Disagree strongly*) to 4 (*Agree strongly*). Higher scores on UPPS-Positive Urgency indicate greater levels of positive urgency. The Cronbach's alpha for both the negative urgency and positive urgency subscales were high (α = 0.88 and 0.93). Scores ranged from 12 to 48 (M NU = 28.52, SD = 7.62; M PU = 24.07, SD = 7.72).

#### Daily EMA assessments for 2 weeks

##### Positive and negative affect

State positive and negative affect was measured at every EMA prompt with the Positive and Negative Affect Scale (PANAS; Watson et al., 1988). This scale is suited for EMA methodology as participants briefly rate discrete emotions at each time point. The positive affect subscale consisted of the following items: alert, inspired, determined, attentive, active. Individuals rated each discrete emotion on a 5-point scale ranging from 1 (*Not at all*) to 5 (*Extremely*). Higher scores on the positive affect subscale of the PANAS indicate greater positive affect levels. The Cronbach's alpha (calculated using the first EMA response from each participant) for the positive affect subscale was fairly high (α = 0.84).

##### Objective binge eating and alcohol use

The behaviors assessed in the EMA protocol included objective binge eating, purging (i.e., self-induced vomiting and/or use of laxatives to limit caloric intake), alcohol use, aggression, and non-suicidal self-injury (NSSI). For the purpose of this study, we used initiation of drinking and objective binge eating as behavioral variables of interest. Participants were asked if they had engaged in any behaviors since their last prompt. For each separate behavior assessed, responses were coded as a 1 if the participant reported engaging in the behavior and were coded as a 0 if the participant reported that they did not engage the behavior. Participants were asked to report on alcohol use, objective binge eating, and all other behaviors at each time point. For alcohol use, responses were dichotomized such that 1 indicated drinking at least some alcohol, and 0 indicated that no alcoholic beverages were consumed.

##### Drinking and eating motives

If participants indicated that they had engaged in a behavior, then they were asked a series of follow up questions. Participants were asked to rate the extent that they had engaged in drinking or binge eating for each of the following reasons: “Because it's fun”; “To feel less depressed, nervous, or stressed”; “To forget about my worries”; “To fit in with others”; “To make a social event more fun”; and “Because I wanted to feel more self-confident and sure of myself.” These items were generated from validated self-report measures of drinking (Cooper, 1994) and eating (Jackson et al., 2003) motives, derived from Cox and Klinger's four factor motivational model of alcohol use (Cox and Klinger, 1988). Participants were asked to rate each item on a scale of one to five. A score of “5” was defined as “Definitely the reason” while a score of “1” was defined as “Not at all the reason.” For the binge eating ratings only, participants were also asked to rate the following motive: “Because I was physically hungry,” on the same scale. If a participant reported that they had consumed alcohol, they were asked if they were alone or with others, how many others, and how many drinks they had consumed.

#### Procedures

The study and associated studies were advertised on two different undergraduate institutions in the Washington DC metro area. After contacting the lab, participants completed an online screener on Qualtrics that included the UPPS- P Impulsive Behavior Scale. If participants met inclusion and exclusion criteria, informed consent was conducted over the phone or in person in the lab. Participants were trained on how to complete the ecological momentary assessment prompts, and were provided with clear definitions for the constructs included in the protocol. Participants were asked to complete one to two practice days of EMA and received feedback from the research team regarding their data during this practice period. Binge eating was defined as consuming an amount of food that would be considered excessive within a 2-h timeframe while experiencing a loss of control. Number of drinks was defined according to the U.S. standard drink (14 g of pure alcohol per drink) and examples were provided. Participants also received a copy of these definitions to refer to throughout the EMA period. This procedure has been utilized in multiple different EMA studies that assessed objective binge eating (e.g., Smyth et al., 2007; Fischer et al., 2018; Wilkinson et al., 2022).

A trained research assistant enrolled participants in EMA and provided feedback on participants' compliance throughout the study. Data were collected through Real-Time Assessment in the Natural Environment (ReTAINE; http://retaine.org/), which allows for web-based collection of EMA data for participants with smartphones. Included in the EMA prompts were assessments of affect, objective binge eating, self-induced vomiting, laxative use, alcohol use, aggressive behaviors, and NSSI engagement. Both studies utilized event-contingent (participants fill out the brief survey after a designated event occurs) and signal-contingent (participants receive a set number of prompts randomly throughout the day) self-report methods. For the signal-contingent prompts, participants received six semi-random prompts throughout the day at randomly selected times around anchor points between 8:30 a.m. and 10 p.m. Participants completed EMA prompts for a 2-week period. Depending upon which university the participant attended, they either received undergraduate research credit or monetary compensation for their participation in the study. Participants were awarded bonus credits or money for completing 85% of their prompts on time.

## Data analysis plan

### Power

*Post hoc* power analyses were conducted with the EMAtools package for R version 4.0.5 (Kleiman, 2017). Power to detect at least a medium effect size with 106 participants, with a 14 day assessment period, six assessments per day, and 75% compliance was 0.80.

In order to test the hypotheses that (1) alcohol use increases odds of objective binge eating and (2) individual differences in NU and PU would moderate the impact of alcohol use on odds of objective binge eating, we used time-lagged models, in which the outcome variable was the first episode of one behavior (e.g., objective binge eating) and the predictors was another behavior (e.g., initiation of alcohol use) earlier in the same day. In other words, was alcohol use a significant predictor of objective binge eating later that same day? We estimated two separate models. In one model, NU (continuous variable) was entered as a main effect in addition to the presence of alcohol use. In the second model, PU (continuous variable) was entered as a main effect. In both models, we also estimated the interaction term of NU or PU by the presence or absence of alcohol use. Outcomes were binary (1 = binge eating present; 0 = binge eating absent) with the reference category set to zero. We did not enter NU and PU in the same model as these variables were highly correlated in our sample, similar to other undergraduate samples, and are considered facets of the same higher order construct (Cyders and Smith, 2007).

Theoretically, trait urgency functions to influence behavior such that individuals with high levels of NU are more likely to behave impulsively when experiencing acute negative affect, and that individuals with high levels of PU are more likely to behave impulsively when experiencing acute positive affect. Therefore, in addition to examining whether or not alcohol use predicted later same day objective binge eating, we wished to examine whether changes in affect during the day significantly predicted initiation of drinking. We hypothesized that positive affect may increase the likelihood that individuals may drink in an emerging adult sample, and that this may be moderated by individual differences in PU, such that individuals high in PU would be more likely to drink if positive affect increased. We also examined whether or not trajectories of positive affect change after initiation of drinking, and whether or not individuals differences in PU moderated the post drinking affect trajectory. We tested the same model with negative affect and NU.

Because EMA data is time series data with multiple data points per day for each participant, the observations are not independent from one another. As such, a generalized linear model is not appropriate, and we instead used generalized estimating equations (GEE) to examine the temporal relationship between positive affect and initiation of drinking. This method allows for examination of the rate and direction of change of positive affect before and after engagement in a behavior through piecewise, linear, quadratic, and cubic functions. The models were based on a gamma distribution with a log link function due to the positive skew of the data. To account for the correlation across repeated observations of each participant, the models included a second-order dependent covariance structure. The GEE models included linear functions (which calculate the rate of change in positive affect before and after a behavior occurs), quadratic functions (which reflect the acceleration of the rate of change in positive affect before and after a behavior occurs), and cubic functions (which represent the further acceleration or dampening of the acceleration in the rate of positive affect). Data from the signal contingent responses for the EMA were used for positive affect ratings and engagement in behaviors, while the event contingent responses were used as a validity check for the signal contingent data. Positive affect trajectories were measured over the course of 4 h before and 4 h after a behavior occurred. If participants engaged in more than one behavior in a day, only the first behavior was included in the analyses to prevent confounding antecedent and consequent positive affect ratings when multiple dysregulated behaviors occurred in a day. If another behavior occurred within the 4-h time span, positive affect ratings after the initial behavior were not included in the analyses up until engagement in the next behavior. We conducted the same analyses with negative affect, initiation of drinking, and NU.

To examine PU as a potential moderator to the relationship between positive affect and initiation of drinking, positive urgency was entered in the GEE model as both a main effect and an interaction term for each of the time components to determine whether the trajectory of positive affect before and after the behavior was dependent on PU scores. Prior to the analysis, the moderator was standardized and mean-centered. We conducted the same series of analyses with NU and negative affect and initiation of drinking.

We also collected EMA data on motives for drinking and eating, and on the environmental context of drinking behavior. We hypothesize that positively valenced drinking experiences may increase the likelihood of binge eating in this age group. Therefore, we examined descriptive statistics regarding the motives and context for drinking episodes that occurred prior to binge eating, and motives for binge eating that occurred following drinking.

## Results

There was a total of 7,821 EMA responses, which represented 1,456.57 separate participant days. The compliance rate throughout the EMA portion was fairly high (76%). Other EMA studies utilizing undergraduate samples have reported lower or similar compliance rates [e.g., 69.23% (Kleiman et al., 2020); 76.5% (Howard and Lamb, 2022)]. During the EMA period, 130 objective binge episodes were reported, and 319 drinking episodes were reported. A total of 37 participants reported an objective binge episode, while a total of 95 participants reported having initiating alcohol use at least once during the EMA period.

### Alcohol use, binge episodes, and positive and negative urgency

We first conducted the time lagged analyses testing the hypotheses that alcohol use increases the likelihood of objective binge eating later in the same day, and that individual differences in PU and NU would increase the likelihood of objective binge eating following alcohol use. Consistent with our hypotheses, initiation of drinking at one time point was a significant predictor of an objective binge episode at the next time point (*B* = 1.96, *p* < 0.0001). Individual differences in PU also had a significant main effect on objective binge eating following initiation of drinking (*B* = 0.89, *p* < 0.023), such that higher scores on PU were associated with increased odds of objective binge eating after alcohol use in the same day. However, there was not a significant interaction of PU and alcohol use on same day objective binge eating (see Table 1). In contrast, individual differences in NU did not have a significant main effect on objective binge eating following alcohol use. However, there was a significant interaction effect, such that higher scores on NU were associated with increased likelihood of objective binge eating if alcohol had already been consumed (see Table 1).

**Table 1:** Time-lagged models of binge eating and initiation of drinking including positive urgency and negative urgency.

**Model term**	**Coefficient**	**SE**	***t***	***p*-value**	**95% CI lower**	**95% CI upper**
**Alcohol use at time 1 and binge eating at time 2 with PU**						
Intercept	−6.98	0.45	−15.57	0.000001	−7.86	−6.10
**Alcohol** **=** **1**	**1.69**	**0.50**	**3.37**	**0.000001**	**0.71**	**2.67**
Alcohol = 0	0					
**zPU**	**0.89**	**0.39**	**2.27**	**0.023**	**0.12**	**1.66**
zPU × alcohol	0.31	0.34	0.90	0.37	−0.36	0.97
**Alcohol use at time 1 and binge eating at time 2 with NU**						
Intercept	−3.42	0.07	−50.86	0.000001	−3.56	−3.29
**Alcohol** **=** **1**	**0.58**	**0.28**	**2.09**	**0.037**	**0.35**	**1.18**
Alcohol = 0	0					
zNU	0.12	0.07	1.71	0.087	−0.02	0.25
**zNU** **×alcohol**	**0.63**	**0.24**	**2.68**	**0.007**	**0.17**	**1.09**

Significant findings (*p* < 0.05) are bolded.

zPU, centered scores on positive urgency; zNU, centered scores on negative urgency; alcohol, 1 indicates the reference category of the presence of initiation of drinking.

### Trajectory of positive and negative affect before and after initiation of drinking and moderation by PU and NU

In contrast to hypotheses regarding positive affect and drinking in emerging adults, there were no significant changes in the linear trajectory of positive affect prior to or following initiation of alcohol use (see Table 2). There was not a significant moderation effect of PU on positive affect and initiation of drinking, nor of NU on negative affect and initiation of drinking.

**Table 2:** Generalized estimating equations (GEE) analysis for trajectory of positive affect before and after initiation of drinking.

**Initiation of drinking**	**Estimate**	**SE**	***p*-value**
Intercept	0.61	0.04	<0.001
Hours	0.004	0.02	0.85
Hours^2^	0.003	0.006	0.57
Hours^3^	<0.001	0.0003	0.41
Hours^*^ Alcohol	0.092	0.04	0.02
Hours^2*^ Alcohol	−0.021	0.009	0.018
Hours^3*^ Alcohol	0.001	0.0004	0.22
Hours^*^ PU	−0.034	0.02	0.09
Hours^2*^ PU	−0.004	0.005	0.40
Hours^3*^ PU	<-0.0001	0.0003	0.86
Hours^*^ Alcohol^*^ PU	−0.007	0.05	0.86
Hours^2*^ Alcohol ^*^ PU	0.013	0.04	0.17
Hours^3*^ Alcohol ^*^ PU	<0.0001	0.0004	0.33

PU, positive urgency; Hours, linear trajectory of positive affect prior to drinking; Hours^2^, cubic trajectory of positive affect prior to drinking; Hours^3^, quadratic trajectory of positive affect prior to drinking. Hours^*^ Alcohol, Hours^2*^ Alcohol, and Hours^3*^ Alcohol, the linear, cubic, and quadratic trajectories of positive affect following initiation of drinking alcohol; Hours^*^ PU, Hours^2*^ PU, and Hours^3*^ PU, the interaction of the linear, cubic, and quadratic trajectories of positive affect prior initiation of drinking alcohol and positive urgency; Hours^*^ Alcohol^*^ PU, Hours^2*^ Alcohol^*^ PU, and Hours^3*^ Alcohol^*^ PU, the interaction of the linear, cubic, and quadratic trajectories of positive affect and positive urgency following initiation of drinking alcohol.

This table represents findings when the moderator (PU) was included in the model. When the moderator was not included in the model at all, there were no significant changes in trajectories of positive affect prior to and following drinking. The interaction of time by PU is not significant prior to nor following initiation of drinking. Therefore, given that the final step of the model in which the moderator is included was not significant, significant findings are not interpreted.

### Descriptive statistics of alcohol use, objective binge eating episodes, and motives

Out of the 319 drinking episodes, 286 (89.66%) occurred with other people. Among those episodes, 154 episodes occurred with five or more people, 58 with three to four other people, and the remainder with one to two other people. The most highly rated motive for drinking was “to have fun” (mean score of 3.62). Thus, the vast majority of drinking episodes were social drinking episodes and motivated by positive emotion enhancement, despite the fact that positive emotion did not change prior to drinking. We also examined descriptive statistics for objective binge episodes that were preceded by alcohol use. Approximately 81% of these binge episodes were preceded by alcohol use that occurred with other people, and 19% were preceded by solitary drinking episodes. Thus, the majority of objective binge episodes following drinking occurred after social drinking episodes. The most highly rated motive for objective binge episodes that occurred following alcohol use was “Because I was physically hungry” (mean score of 2.81). This is consistent with experimental studies that indicating that hunger ratings increase following alcohol consumption (e.g., Yeomans, 2010).

## Discussion

We posit that one potential developmental pathway to objective binge eating may be alcohol use itself, which is often associated with social facilitation, mood enhancement, and positive emotional experiences in emerging adults. In this sample of emerging adults, initiation of drinking alcohol was a significant predictor of objective binge eating later in the same day. Individual differences in the tendency to act impulsively while experiencing positive affect were also significant predictors of objective binge eating after initiation of drinking. However, positive affect did not increase prior to initiation of drinking, and neither did negative affect. The vast majority of drinking episodes that occurred prior to objective binge eating were social drinking episodes. Additionally, individual differences in NU interacted with initiation of alcohol use, such that individuals with higher scores on NU who also drank alcohol had increased odds of later objective binge eating on the same day.

The primary hypothesis that alcohol use increases the likelihood of later same day binge eating was supported. We also posited that in an emerging adult sample, alcohol use would be associated with positive affect. In this sample, changes in levels of affect (positive or negative) were not significantly associated with initiation of drinking. However, the majority of drinking episodes occurred in the presence of other people, and were associated with the motive “to have fun.” This suggests that perhaps alcohol use functioned to increase positive affect in social contexts, not to amplify positive affect that had already increased. The belief or outcome expectancy that alcohol will increase positive emotional experiences is a predictor of drinking itself (e.g., Creswell, 2021).

This study did not examine increases in craving or changes in inhibitory control. However, experimental literature in the alcohol field suggests that enhanced reward seeking and decreased inhibitory control may lead to continued drinking and eating in the presence of moderate doses of alcohol. There was a main effect of PU on binge eating and an interaction of NU with alcohol on binge eating, and these traits are associated with decreased inhibitory control in the presence of emotion (Johnson et al., 2020). Thus, it is possible that alcohol consumption itself both decreased inhibitory control and made those with high levels of urgency more vulnerable to impulsive eating, especially if alcohol consumption amplified affect. However, due to the nature of the EMA data collection in this study, we were not able to examine how the consumption of alcohol itself may have impacted affective trajectories prior to objective binge eating. Additionally, other studies have examined individual differences in reward seeking and fun seeking, and found that these traits are positively associated with binge eating indirectly via expectancy development (Schell et al., 2019). We did not assess these traits in this study. Thus, it is also possible that alcohol contributed to binge eating via amplification of reward responsiveness in individuals with these traits.

## Limitations and future directions

Some of the limitations of the study are inherent in the sampling method used. We provided feedback and training on the definitions of objective binge eating, but cannot corroborate the reports obtained in EMA. Additionally, there are several aspects of alcohol use to consider when collecting data on this behavior via EMA. First, once someone has begun drinking, it may be difficult to obtain accurate reports of how much they consume or their behavior following drinking, including eating behavior. It also may be difficult for a person to report on loss of control over eating if they were intoxicated. We were not able to provide breathalyzer analysis to corroborate the quantity of alcohol consumed. We specifically chose to consider initiation of alcohol use in this study, due to some of these considerations. However, in future studies, it would be helpful to assess how blood alcohol concentration prior to binge eating, as experimental studies indicate that the dosage of alcohol consumed has an impact on reward seeking behavior and loss of inhibitory control. Related to these points, the assessment period ended at 10:00 p.m. each day. Some individuals may have initiated drinking past 10:00 p.m. In this EMA protocol, participants were randomly signaled in designated blocks of time during the day. Therefore, it was difficult to examine changes in affect immediately following alcohol use and immediately prior to binge eating in the same episode. For example, if a person responded to a prompt at 8:00 p.m. and reported drinking, and then responded to the next prompt and reported binge eating, it is possible that the binge eating occurred immediately after their response to the previous prompt and within the same hour that alcohol use had occurred. It would be informative to collect more fine grained analysis of changes in affect between these two behaviors.

Additionally, we focused our analyses on objective binge eating. It is possible that increases in positive emotion throughout the day or alcohol use is associated with other eating behaviors, such as snacking (Bongers et al., 2013; Evers et al., 2013), as opposed to objective binges. Future research could also assess eating behavior other than binge eating via EMA. It is possible that increased snacking may lead to subjective experiences of loss of control, which is associated with the development of disordered eating (Byrne et al., 2019). Finally, although we assessed several contextual factors associated with alcohol use, we did not ask parallel questions regarding the binge episodes. For example, we do not know if the binge episodes occurred in the presence of other people or alone. Loss of control over eating while in a social context may be driven by different individual difference factors than solitary loss of control over eating.

Our sample was not a clinical sample of individuals with EDs, and attempted restraint over eating is associated with increased likelihood of objective binge eating (Wilkinson et al., 2022). It may be that some individuals with high levels of restrained eating, such as those with BN, may restrict eating prior to drinking, increasing vulnerability to binge eating. However, it also may be the case that some individuals with high levels of impulsivity or disinhibition are more likely to engage in multiple impulsive behaviors on the same day, and thus binge eat prior to drinking. There may be different mechanisms that lead to binge eating prior to alcohol use and alcohol use prior to binge eating. One potential direction for future research is to examine whether or not urgency interacts with restraint to predict different temporal patterns of binge eating and alcohol use. Future research should also examine the association between positive urgency and inhibitory control to better understand the role this trait may play in alcohol use and binge eating, beyond the effects of alcohol/intoxication alone. Finally, it is important to consider the developmental timing of the onset of these behaviors. It is possible that social drinking and positive emotion dysregulation increases risk for binge eating during this specific developmental period, when many emerging adults experience a peak in drinking behavior (Grant et al., 2015).

In summary, alcohol use in this emerging adult sample was a significant predictor of same day binge eating. The alcohol use primarily occurred during social situations and was motivated by fun-seeking. This suggests that there are additional pathways to binge eating beyond increases in negative emotion, and it highlights the importance of considering co-occurring behavior patterns when examining risk for this behavior.

## Data availability statement

The raw data supporting the conclusions of this article will be made available by the authors, without undue reservation.

## Ethics statement

The studies involving human participants were reviewed and approved by George Mason University Institutional Review Board. The patients/participants provided their written informed consent to participate in this study.

## Author contributions

SF conceptualized the paper, wrote the primary draft, and conducted analyses. JW conducted analyses, collected data, and edited the manuscript. LM contributed to writing and editing. LB collected data and contributed to analyses. RF contributed to analyses and edited the manuscript. JN edited the manuscript. All authors contributed to the article and approved the submitted version.
